# Association Between Socio-Political Stress, Perceived Stress, and Bruxism-Related Clinical Findings Among Iranian University Students Studying in Turkey: A Cross-Sectional Study

**DOI:** 10.3390/healthcare14193224

**Published:** 2026-09-30

**Authors:** Ece Yılmazkasapoğlu Türkay, Ayşe Nur Parlakyıldız Gökçe, Kübra Yıldız Domaniç

**Affiliations:** 1 Department of Pediatric Dentistry, Faculty of Dentistry, Istanbul Atlas University, Istanbul 34403, Turkey; aysenur.parlakyildiz@gmail.com; 2 Department of Prosthodontics, Faculty of Dentistry, Istanbul Atlas University, Istanbul 34403, Turkey; kubra.domanic@atlas.edu.tr

**Keywords:** bruxism, stress, psychological, students, emigrants and immigrants, cross-sectional studies, Iran

## Abstract

**Highlights:**

**What are the main findings?**

- Socio-political stress was positively associated with perceived stress but was not associated with bruxism-related clinical findings.
- Bruxism-related clinical findings were present in 55.8% of participants, while older age and moderate/high caffeine intake were associated with greater odds of these findings.

**What are the implications of the main findings?**

- Perceived stress alone may not be an adequate indicator of bruxism risk; clinical and behavioral factors should also be considered during assessment.
- Home-country-related socio-political stress may contribute to the psychological burden of international students, supporting the need for culturally sensitive psychosocial screening and support.

**Abstract:**

**Background**: Students who study abroad frequently contend with several stressors at once—among them academic workload, adjustment to an unfamiliar cultural setting, and worries over political events unfolding in their home countries. Because psychological stress has been linked to bruxism, the present study set out to determine whether socio-political stress and perceived stress were associated with bruxism-related clinical findings among Iranian university students studying in Turkey. **Methods**: This cross-sectional pilot study included 113 Iranian university students. Socio-political stress was assessed using an eight-item researcher-developed module, and perceived stress was measured with the PSS-10. Bruxism-related clinical findings were evaluated by two blinded examiners using a predefined clinical composite. Associations were examined using correlation analyses and fully adjusted logistic regression. **Results**: Bruxism-related clinical findings were present in 63 participants (55.8%), with excellent inter-examiner agreement for the primary clinical composite (κ = 0.982). Socio-political stress was positively associated with perceived stress (Spearman’s rho = 0.339, 95% CI: 0.16–0.49, *p* < 0.001), but neither socio-political stress nor PSS-10 score was independently associated with bruxism-related clinical findings in the fully adjusted model. Older age (OR = 1.483, 95% CI: 1.203–1.828, *p* < 0.001) and moderate/high caffeine intake (OR = 4.12, 95% CI: 1.54–11.02, *p* = 0.005) were associated with higher odds of bruxism-related clinical findings. **Conclusions**: Socio-political stress was associated with perceived stress but not with bruxism-related clinical findings. Older age and moderate/high caffeine intake were associated with higher odds of these findings.

## 1. Introduction

Psychological stress is recognized as an important factor influencing both general health and oral health. University students are particularly vulnerable to stress due to academic demands, financial concerns, and social adaptation challenges. International students face extra stresses in addition to the difficulties faced by domestic students. Aside from their academic obligations, they frequently have to deal with being away from their families, acclimating to a new culture, and learning a foreign language. These pressures may be exacerbated by worries about social and political changes in their home countries. Prior studies have demonstrated that international students’ mental health can be negatively impacted by geopolitical instability, economic uncertainty, and concerns about the security and welfare of family members [1,2].

The degree to which people consider the responsibilities of their everyday life to be overwhelming or challenging to handle is reflected in perceived stress. One of the most often used tools for evaluating psychological stress in behavioural and epidemiological research is the Perceived Stress Scale (PSS), which was developed by Cohen and associates [3]. Numerous psychological and physiological effects, such as anxiety, depression, sleep difficulties, and physical symptoms of stress, have been associated with higher perceived stress scores [4]. University students are typically thought to be more susceptible to high levels of perceived stress due to the demands of their studies and significant life changes.

Repetitive jaw-muscle activity, such as clenching or grinding teeth and/or bracing or thrusting the mandible, are symptoms of bruxism. According to a recent global consensus, bruxism is a behavior that can happen while you are awake or while you are sleeping, rather than a disorder in and of itself [5,6]. Psychological elements, particularly stress and anxiety, are thought to play a significant role in its multiple and complicated etiology [7,8]. Stress is positively correlated with bruxism-related symptoms in a variety of populations, including college students, according to several studies [9]. However, it is still unknown how strong and constant this link is in various cultural and demographic circumstances.

Iranian students form a large and important group among international students in Turkey. In recent years, an increasing number of young Iranians have pursued higher education abroad, a trend linked to economic pressures and recurring periods of social and political instability in Iran. Turkey has become a preferred destination for this population because of its geographic proximity, comparatively lower tuition costs, and more accessible admission and residency procedures. On the other hand, most Iranian students still maintain strong ties to their country: they remain connected through family, regular exposure to local news and social media, and ongoing financial or administrative responsibilities. Consequently, even as students adapt to university life in Turkey, events in Iran can continue to affect their psychological well-being. Most earlier research has focused on international students from more politically stable countries; because of this, few studies have examined the specific stress pattern that can arise when migration stress overlaps with ongoing socio-political concerns [10].

There is growing interest in the psychosocial causes of bruxism, but little is known about how socio-political stress may contribute to bruxism-related behaviors. Iranian students abroad may face specific stressors, such as economic sanctions, geopolitical tensions, and worry about family members at home [11]. It is still unclear whether these extra stressors are linked to a higher rate of bruxism-related clinical findings. Therefore, this cross-sectional study aimed to examine the relationships between socio-political stress, perceived stress, and bruxism-related clinical findings among Iranian university students studying in Turkey. To our knowledge, this is the first study to examine socio-political stress specifically, as distinct from general perceived stress, in relation to bruxism-related clinical findings, reflecting the hypothesis that home-country political and economic instability constitutes a qualitatively distinct psychological burden for international students.

## 2. Materials and Methods

The primary objective of this study was to determine whether perceived stress is associated with bruxism-related clinical findings among Iranian university students studying in Turkey. Secondary objectives were to determine whether socio-political stress is associated with these findings, and whether socio-political stress and perceived stress are correlated with one another in this population. The research was designed as a descriptive and analytical cross-sectional survey study.

### 2.1. Study Setting and Timeline

This research was conducted among Iranian dental students at a single institution (Faculty of Dentistry, Istanbul Atlas University) in Turkey. Data collection was carried out at the Faculty of Dentistry, using a convenience sampling method based on voluntary participation, with the sociodemographic and stress questionnaires completed either on paper or via an equivalent online form. Data collection began following ethics committee approval, which was sought from the Non-Interventional Clinical Research Ethics Committee (approval date: 23 March 2026/approval no: 26/03) and the study was carried out in accordance with the code of ethics in the Declaration of Helsinki. Data collection for this cross-sectional study was carried out between April 2026 and June 2026, after ethical approval had been obtained. The reference to the 2025–2026 academic year describes the academic year in which participants were enrolled at the time of data collection, at the Faculty of Dentistry.

### 2.2. Study Population and Sample

The study population consisted of Iranian dental students enrolled in the Turkish-language undergraduate dental program at the Faculty of Dentistry, Istanbul Atlas University, Istanbul, Turkey. Institutional records identified 197 Iranian dental students registered in the Faculty, of whom 154 were actively enrolled at the time of recruitment; 43 students who were not actively enrolled were therefore outside the recruitment population. Among the 154 active students, 31 were first-year students and were excluded from the sampling frame because they had not yet commenced clinical training and were not routinely present in the clinical/faculty setting where recruitment and data collection were conducted. The remaining 123 students in academic years 2–5 were eligible and invited to attend a scheduled appointment. Of these, 113 attended, provided written informed consent, and completed both the questionnaire and clinical examination; 10 did not participate. The participation rate among eligible students was therefore 91.9% (113/123). Sampling was based on voluntary participation and was classified as convenience sampling.

**Figure 1:**
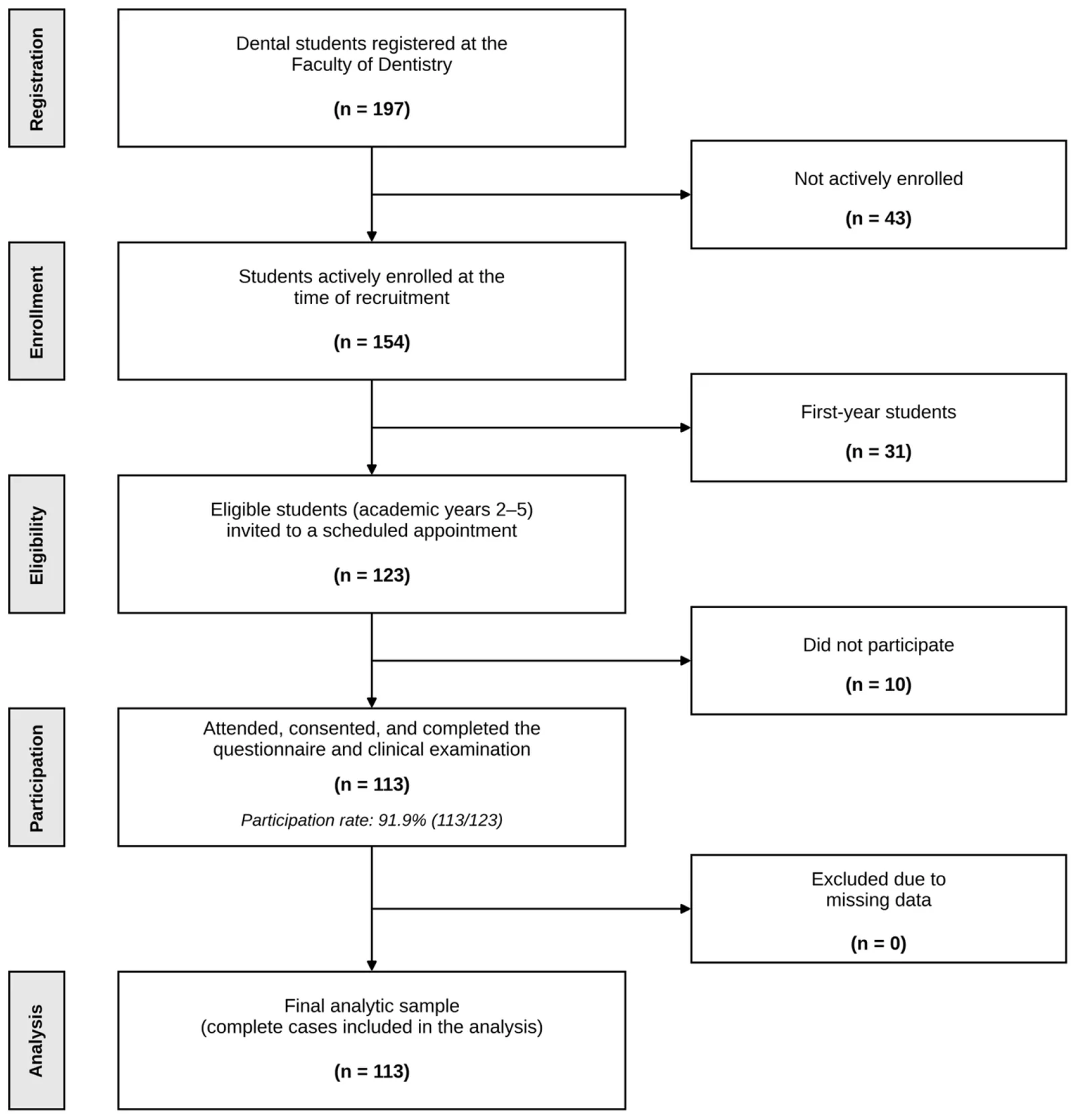
Participant recruitment and analysis flow. The participation rate among eligible students in academic years 2–5 was 91.9% (113/123).

Participant recruitment, eligibility, participation, and inclusion in the final analysis are summarized in Figure 1.

The required sample size was calculated a priori using G*Power 3.1.9.7. Because the primary outcome was binary, the calculation was performed using the z tests family and the logistic regression procedure. A two-tailed test, α = 0.05, 80% power, an expected odds ratio of 1.50, and an outcome probability of 0.50 under the null hypothesis were specified. Under these assumptions, the minimum required sample size was approximately 85 participants. The final analytic sample included 113 participants, with 63 outcome events. Therefore, the achieved sample size exceeded the minimum requirement, although the multivariable models were kept parsimonious because of the limited number of outcome events.

**Inclusion criteria:** Being an Iranian citizen; currently enrolled in undergraduate or graduate education at a university in Turkey; being 18 years of age or older; voluntarily agreeing to participate and providing consent; completing the questionnaire in full.

**Exclusion criteria:** Being under 18 years of age; not being an Iranian citizen; not being a university student in Turkey; incomplete or incorrectly completed questionnaires; refusal to participate or withdrawal from the study.

### 2.3. Data Collection Instruments

Data were collected through a questionnaire form prepared by the researchers. The first page of the form was designed to determine participants’ sociodemographic characteristics, including age, gender, faculty and academic year, length of stay in Turkey, living arrangements, cohabitants, presence of first-degree relatives in Iran, and primary source of income. This section was used to describe participants’ general characteristics and to assess sociodemographic variables potentially associated with stress levels.

The second page of the questionnaire was prepared by the researchers to assess the psychological effects of current socio-political developments in Iran on Iranian students studying in Turkey. These developments were considered within the context of the ongoing socio-political and economic instability in Iran during the period immediately preceding and throughout data collection in early 2026, including broader social, political, and economic uncertainties that could potentially affect students’ psychological well-being and stress levels. The first scale consisted of 8 items assessing participants’ socio-political stress levels experienced over the past month. Items were rated on a 5-point Likert scale (0 = Strongly disagree, 1 = Disagree, 2 = Undecided, 3 = Agree, 4 = Strongly agree). This scale assessed indicators of socio-political stress such as concern about family safety, financial uncertainty, anxiety about being unable to continue education, increased social media and news monitoring, and sleep and concentration problems. Higher scores indicated higher levels of stress related to socio-political developments.

The Perceived Stress Scale (PSS-10) was also included on the second page of the questionnaire to assess participants’ general stress levels [12]. The scale was originally developed by Cohen, Kamarck, and Mermelstein [3] to measure the degree to which individuals perceive events in their lives as stressful, evaluating stress experienced over the previous month. The Turkish validity and reliability study of the scale was conducted by Eskin, Harlak, Demirkıran, and Dereboy (2013) [12]. The scale consists of 10 items rated on a 5-point Likert scale (0 = never, 1 = almost never, 2 = sometimes, 3 = fairly often, 4 = very often). Items 4, 5, 7, and 8 are reverse-scored prior to analysis. The scale has a unidimensional structure and measures an individual’s perceived stress level. Total scores range from 0 to 40, with higher scores indicating higher perceived stress. In the Turkish adaptation study, the Cronbach’s alpha reliability coefficient of the scale was reported as 0.82. The scale has been widely used in different age groups, particularly among university students and adults.

On the same page, participants were also directed to a brief assessment developed by the researchers to evaluate the presence of bruxism. This section included questions addressing symptoms of bruxism such as teeth clenching/grinding behavior, morning jaw fatigue, headache, tooth sensitivity, and noticeable tooth wear. Some items were rated on a 5-point Likert scale (0 = never, 4 = very often), while others were answered as yes/no. Behavioral factors potentially associated with bruxism, such as smoking and caffeine consumption, were also assessed. Data obtained from this section were used to determine the presence of possible bruxism symptoms and to examine their relationship with stress levels. On the third page of the questionnaire, participants underwent a face-to-face clinical examination performed independently by two examiners. The clinical examination recorded tooth-wear scores using the Basic Erosive Wear Examination (BEWE) index, scored separately for the anterior and posterior sextants; the presence of soft-tissue or muscle-related findings (e.g., linea alba, cheek-biting marks, tongue scalloping, or masseter hypertrophy); muscle palpation tenderness; and temporomandibular disorder (TMD)-related findings, including jaw pain or locking, deviation on mouth opening, and joint sounds. The BEWE was selected as a standardized, widely validated, and simple-to-apply index for quantifying cumulative tooth-surface loss, used here as a practical proxy indicator of possible attritional wear rather than as a bruxism-specific diagnostic instrument in its own right. Examiners were instructed to score wear patterns consistent with mechanical attrition—flattened cusp tips or facets, without the smooth, non-anatomic, and often concave surface loss typical of chemical or acid erosion—and to exclude wear with a clear participant-reported dietary or gastric-reflux etiology; nonetheless, erosive and attritional wear cannot always be fully distinguished on clinical grounds alone, which we acknowledge as a limitation (Section 4). Examiner training and calibration: Examiner training and calibration were conducted prior to data collection. The clinical assessments were performed independently by a specialist in pediatric dentistry and a prosthodontist. Before the main study, both examiners independently evaluated the same 20 calibration cases using the predefined clinical assessment criteria, including the BEWE index, soft-tissue and muscle-related findings, muscle palpation tenderness, and temporomandibular disorder-related findings. Their assessments were subsequently compared, and any discrepancies were discussed until consensus was reached to standardize the interpretation and application of the clinical criteria. These calibration cases were not included in the final study sample. Intra-examiner reliability was not assessed because the same cases were not re-evaluated after a separate time interval. For this study, a participant was classified as having a bruxism-related clinical finding if at least one of the following clinically examined components was present: a BEWE tooth-wear score ≥1 on any surface, at least one soft-tissue or muscle-related finding, muscle palpation tenderness ≥ grade 1, or at least one temporomandibular disorder-related finding. Self-reported clenching or grinding, described above, was recorded and analyzed separately and was not included in the clinical composite. Examiners recorded findings independently, without access to each other’s ratings or to participants’ self-reported questionnaire responses, on separate case-report forms; the two examiners’ composite classifications were subsequently compared for the inter-examiner reliability analysis reported in Section 3.3. It should be noted that tooth wear, soft-tissue changes, muscle tenderness, and TMD-related findings are not specific to current bruxism and may reflect cumulative or unrelated exposures; this composite therefore indexes clinical signs associated with possible bruxism activity rather than constituting a definitive bruxism diagnosis.

### 2.4. Data Collection Procedure

Data were collected in a single supervised session at the Faculty of Dentistry. Participants were first provided with written information about the study, and voluntary informed consent was obtained. Participants then completed the sociodemographic questionnaire, the socio-political stress module, and the PSS-10, either on paper or via an equivalent online form, independently and anonymously; the survey was designed so that each participant could complete it only once. Immediately afterward, participants underwent a face-to-face, non-invasive clinical examination performed independently by the two blinded examiners described in Section 2.3. Questionnaire responses and clinical findings were linked using a sequential, non-identifying study ID assigned at enrollment, with no names or contact details retained in the analytic dataset. Because the full protocol was completed within a single supervised session, no participant could re-enter the survey independently, preventing duplicate entries. No therapeutic or diagnostic intervention beyond this non-invasive clinical screening was performed during the data collection process.

### 2.5. Statistical Analysis

Statistical analyses were performed using Jamovi version 2.7 and R version 4.5. All analyses were conducted on the final analytic sample of 113 participants. Continuous variables were summarized as mean ± standard deviation, median, and minimum–maximum values, whereas categorical variables were presented as frequencies and percentages. The normality of continuous variables was assessed using the Shapiro–Wilk test together with visual inspection of histograms and Q–Q plots. Normality testing was not applied to binary or categorical variables. A *p*-value < 0.05 was considered statistically significant.

Internal consistency of the researcher-developed socio-political stress module and the PSS-10 was evaluated using Cronbach’s alpha, corrected item-total correlations, and alpha-if-item-deleted values. For the PSS-10, reverse scoring of items 4, 5, 7, and 8 was verified before calculating the total score. Because the observed internal consistency of the PSS-10 was lower than expected in this sample, analyses based on the PSS-10 total score were interpreted as exploratory.

For the socio-political stress module, exploratory dimensionality was examined using principal component analysis. Sampling adequacy was assessed using the Kaiser–Meyer–Olkin statistic, and Bartlett’s test of sphericity was used to determine whether the item correlation matrix was suitable for component extraction. Component retention was evaluated using eigenvalues greater than 1, inspection of the scree plot, and parallel analysis. Item loadings were reported descriptively. Because this module was newly developed and evaluated in the same analytic sample, these psychometric analyses were considered exploratory and were not interpreted as evidence of full construct validation.

Because the main continuous variables departed from normality, between-group comparisons according to bruxism-related clinical findings status were performed using the Mann–Whitney U test as the primary analysis. Corresponding parametric comparisons were retained only as supplementary sensitivity analyses where relevant. The association between socio-political stress and perceived stress was examined using Spearman’s rank correlation coefficient as the primary correlation analysis, because both variables departed from normality. Pearson correlation analysis was considered only as a supplementary sensitivity analysis.

Binary logistic regression analyses were performed to examine variables associated with bruxism-related clinical findings status. The primary outcome was coded as absent or present bruxism-related clinical findings. The main exposure variables were socio-political stress total score and PSS-10 total score. Age was entered as a continuous covariate. Gender was reported descriptively and evaluated as a candidate covariate in supplementary regression analyses. Smoking status and daily caffeine intake were evaluated as behavioral covariates. To avoid assuming equal log-odds intervals across ordinal response categories, smoking and caffeine variables were also examined using collapsed categorical indicator variables where appropriate. Odds ratios, 95% confidence intervals, and *p*-values were reported.

The multivariable model was specified parsimoniously because 63 participants had the primary outcome. This number of outcome events was considered when limiting the number of predictors entered simultaneously into the model. Model diagnostics were examined for the logistic regression analyses. The linearity of continuous predictors in the logit was assessed using the Box–Tidwell approach. Multicollinearity was evaluated using variance inflation factors. Model calibration was assessed using the Hosmer–Lemeshow goodness-of-fit test, and model discrimination was summarized using the C-statistic/area under the receiver operating characteristic curve. Influential observations were examined using Cook’s distance. Because there were no missing data for the variables included in the regression models, complete-case exclusion was not required.

## 3. Results

### 3.1. Participant Characteristics

A total of 113 Iranian university students studying in Turkey were included in the final analysis, with no missing data for age, duration of residence in Turkey, academic year, socio-political stress total score, PSS-10 total score, or bruxism-related clinical findings status. Participants’ mean age was 24.1 ± 3.33 years (median 23, range 20–35), and they had lived in Turkey for a mean of 50.1 ± 19.0 months (median 49, range 25–132). The mean academic year was 3.29 ± 1.03, distributed as 16 fifth-year, 33 fourth-year, 32 third-year, and 32 second-year students.

Mean scores were 34.0 ± 7.44 (median 36, range 0–40) for socio-political stress and 25.6 ± 4.71 (median 24, range 12–37) for PSS-10. Bruxism-related clinical findings were present in 63 participants (55.8%) and absent in 50 (44.2%). The Shapiro–Wilk test indicated that the continuous variables, including age, duration of residence in Turkey, academic year, socio-political stress score, and PSS-10 score, departed from normality; therefore, non-parametric methods were used as the primary approach for between-group comparisons involving continuous variables. The participants’ demographic and baseline characteristics are summarized in Table 1.

### 3.2. Reliability and Dimensional Structure of the Study Instruments

The researcher-developed socio-political stress module showed good internal consistency (Cronbach’s α = 0.834). Corrected item-total correlations ranged from 0.47 to 0.74, and deletion of individual items did not materially improve the overall reliability coefficient. The Kaiser–Meyer–Olkin measure of sampling adequacy was 0.811, and Bartlett’s test of sphericity was significant (χ^2^ = 398.07, df = 28, *p* < 0.001), indicating that the item correlation matrix was suitable for exploratory component analysis. Principal component analysis of the eight socio-political stress items showed a dominant first component with an eigenvalue of 4.06, explaining 50.3% of the total variance. All eight items loaded positively on the first component, with loadings ranging from 0.56 to 0.81. Although the Kaiser eigenvalue-greater-than-one criterion suggested two components, inspection of the scree plot and parallel analysis supported retention of one dominant component. Therefore, the module was interpreted as showing exploratory evidence of internal coherence rather than full construct validation.

The PSS-10 showed lower-than-expected internal consistency in the present sample (Cronbach’s α = 0.562). One reverse-scored item showed a near-zero corrected item-total correlation (r = 0.01), and deletion of this item increased alpha only modestly to 0.60. Therefore, the PSS-10 total score was retained for consistency with the prespecified analysis, but findings involving this score were interpreted cautiously. Internal consistency results are summarized in Table 2.

### 3.3. Inter-Examiner Reliability of Bruxism-Related Clinical Findings

All 113 participants were independently examined by two trained, calibrated examiners blinded to each other’s findings, to self-reported responses, and to study hypotheses (one item, joint sound, had a single missing value for the second examiner; *n* = 112 for that comparison). Inter-examiner agreement (Cohen’s kappa, weighted for ordinal and unweighted for binary variables) was excellent to almost perfect overall (κ = 0.900–1.000). Granular BEWE tooth-wear scores showed excellent but not absolute agreement (κ = 0.958–0.991; 1–5 single-grade discrepancies per region of 113); muscle palpation scores ranged from κ = 0.938–0.994, and jaw pain/locking showed the lowest agreement observed (κ = 0.900; 4 discordant cases). For the composite bruxism-related clinical finding (primary outcome), agreement remained excellent (κ = 0.982, 99.1%; 1 discordant case). Full results are in Table 3.

### 3.4. Sociodemographic and Background Characteristics

Most participants reported having a first-degree relative in Iran (*n* = 100, 88.5%), while 13 (11.5%) did not. Among those with a first-degree relative in Iran, 46 (86.8%) reported difficulty communicating with them, while 7 (13.2%) did not. The most common source of income was family support (*n* = 99, 87.6%); 9 participants (8.0%) reported both family support and employment, and 5 (4.4%) relied on employment alone. Regarding living arrangements, 48 participants (42.5%) lived alone, 33 (29.2%) lived with friends, and 32 (28.3%) lived with family.

Tobacco use was reported by a minority of participants: 67 (59.3%) reported no use, 17 (15.0%) rare/social use, 21 (18.6%) 1–10 cigarettes/day, and 8 (7.1%) more than 10 cigarettes/day. Daily caffeine intake was absent in 7 (6.2%), low in 53 (46.9%), moderate in 38 (33.6%), and high in 15 (13.3%) participants. Following recent developments in Iran, 62 participants (54.9%) reported an increase in related habits, 30 (26.5%) reported a marked increase, and 21 (18.6%) reported no change.

### 3.5. Self-Reported and Bruxism-Related Clinical Findings

Self-reported bruxism-related symptoms were common. Daytime tooth clenching was reported at varying frequencies, with 83 participants (73.5%) reporting at least some degree of daytime clenching. Regarding sleep-related bruxism, 26 participants (23.0%) reported being told they clenched or ground their teeth during sleep, 41 (36.3%) reported no such history, and 46 (40.7%) did not know. Morning jaw fatigue or headache was reported by 75 participants (66.4%) at some level of frequency. Tooth sensitivity was reported by 41 participants (36.3%), and self-perceived tooth cracks or wear by 28 (24.8%).

Clinical tooth wear (BEWE ≥ 1) was present in 89 participants (78.8%), with anterior wear more common than posterior wear. At least one soft tissue or muscle-related finding (linea alba, cheek biting marks, tongue scalloping, or masseter hypertrophy) was present in 82 participants (72.6%). Muscle palpation tenderness of at least grade 1 was present in 72 participants (63.7%). Temporomandibular disorder-related findings—jaw pain/locking, deviation during mouth opening, or joint sounds—were present overall in 43 participants (38.1%). Combining these self-reported and clinical criteria, bruxism-related clinical findings were present in 63 participants overall (55.8%). Detailed frequencies are presented in Table 4.

### 3.6. Comparison of Stress Scores According to Bruxism-Related Clinical Findings Status

Socio-political stress and PSS-10 scores were compared between participants with and without bruxism-related clinical findings. Participants with bruxism-related clinical findings had a mean socio-political stress score of 33.7 ± 7.64 (median 37.0), compared with 34.3 ± 7.25 (median 36.0) among participants without bruxism-related clinical findings; this difference was not statistically significant (Mann–Whitney U = 1542, *p* = 0.849). Similarly, PSS-10 scores did not differ significantly between participants with bruxism-related clinical findings (25.5 ± 4.96, median 25.0) and those without (25.7 ± 4.41, median 24.0) (Mann–Whitney U = 1558, *p* = 0.921). These results indicate that neither socio-political stress nor perceived stress scores were significantly higher among participants with bruxism-related clinical findings compared with those without. Results are summarized in Table 5.

### 3.7. Correlation Between Socio-Political Stress and Perceived Stress

A statistically significant positive correlation was observed between socio-political stress total score and PSS-10 total score using Spearman’s rank correlation analysis (rho = 0.339, *p* < 0.001). This finding indicates that higher socio-political stress was associated with higher perceived stress in this sample.

### 3.8. Logistic Regression Analysis

Binary logistic regression analysis was performed to examine whether socio-political stress and perceived stress were independently associated with bruxism-related clinical findings. The primary outcome was the presence of bruxism-related clinical findings. The fully adjusted model included socio-political stress total score, PSS-10 total score, age, sex, smoking status, and daily caffeine intake. To avoid assuming equal log-odds intervals across ordinal response categories, smoking status and daily caffeine intake were entered using collapsed categorical indicator variables. Smoking status was coded as current smoker versus non-smoker, and daily caffeine intake was coded as moderate/high intake versus none/low intake.

In the fully adjusted model, socio-political stress total score was not significantly associated with bruxism-related clinical findings (OR = 0.972, 95% CI: 0.906–1.044, *p* = 0.442). PSS-10 total score was also not significantly associated with the outcome (OR = 0.966, 95% CI: 0.868–1.075, *p* = 0.526). Age remained significantly associated with higher odds of bruxism-related clinical findings; each additional year of age was associated with higher odds of presenting these findings (OR = 1.483, 95% CI: 1.203–1.828, *p* < 0.001). Sex was not significantly associated with the outcome (female vs. male: OR = 1.723, 95% CI: 0.639–4.644, *p* = 0.282). Moderate/high caffeine intake was associated with higher odds of bruxism-related clinical findings compared with none/low caffeine intake (OR = 4.12, 95% CI: 1.54–11.02, *p* = 0.005), whereas current smoking was not significantly associated with the outcome compared with non-smoking status (OR = 1.43, 95% CI: 0.58–3.51, *p* = 0.431).

Model diagnostics did not indicate major violations of logistic regression assumptions. The Box–Tidwell tests for socio-political stress total score, PSS-10 total score, and age were non-significant, supporting the linearity of continuous predictors in the logit. Multicollinearity was not problematic, with all variance inflation factors ≤1.34. The Hosmer–Lemeshow test indicated acceptable calibration (χ^2^ = 12.84, df = 8, *p* = 0.117), and the C-statistic was 0.797 (bootstrap 95% CI: 0.705–0.878), indicating acceptable discrimination. No observation exceeded Cook’s distance of 1.0, although six observations exceeded the conservative 4/n screening threshold. There were no missing data for the model variables. The results of the logistic regression analysis are summarized in Table 6, and model diagnostics are provided in Supplementary Table S1.

## 4. Discussion

This study investigated the relationship between socio-political stress, perceived stress, and bruxism-related clinical findings among Iranian university students studying in Turkey. Contrary to our initial hypothesis, perceived stress (PSS-10 score) was not significantly associated with bruxism-related clinical findings status in any model, and neither socio-political stress nor perceived stress scores differed significantly between students with and without bruxism-related clinical findings.

In this study, we did not find a significant link between perceived stress and bruxism-related clinical findings among Iranian university students studying in Turkey. This result is different from earlier studies, which found a positive link between perceived stress and bruxism symptoms in university students and young adults [9,13]. There are several possible reasons for this difference.

First, this was a pilot study with a very small and similar (homogeneous) sample. This may have made it harder to detect small correlations. The very low McFadden’s R^2^ of the uncorrected model (0.00064) supports this idea. It shows that perceived stress explained only a small part of the differences in bruxism-related clinical findings in this sample. Also, bruxism was not measured with instruments such as polysomnography or electromyography. Instead, it was measured using a combination of self-reported symptoms and a standard clinical exam. Clinical signs, such as tooth wear and muscle-related findings, may not respond quickly to stress felt at one point in time, because these signs usually build up over a longer period. Bruxism is a complex condition. Because of this, other factors—such as anxiety, symptoms of depression, sleep quality, coping strategies, and occlusal (bite-related) features—may matter more than perceived stress alone [14]. The quality of the measurement tools themselves may have also played a role.

In this sample, the Turkish PSS-10 showed lower internal consistency (Cronbach’s α = 0.562) than earlier reports had indicated [12]; notably, one reverse-scored item had an almost negligible corrected item–total correlation (r = 0.01), which suggests that participants may not have interpreted the reverse-worded items consistently. Because reduced reliability tends to weaken observed associations, this may partly explain why no statistically significant relationship emerged between perceived stress and bruxism-related clinical findings. The researcher-developed socio-political stress module, on the other hand, performed satisfactorily on both counts, with good internal consistency (Cronbach’s α = 0.834) and a clear single-factor structure accounting for 50.3% of the total variance—making it less likely that measurement error explains the lack of association between socio-political stress and bruxism-related clinical findings.

Socio-political stress was not linked to bruxism-related clinical findings, yet it correlated moderately and positively with perceived stress (Spearman’s rho = 0.339, *p* < 0.001). This is in line with earlier evidence that international students can carry a heavier psychological burden when political instability, economic uncertainty, or concern for family persists in their home countries [15]. Taken together, these findings are consistent with, but do not independently establish, the construct validity of the socio-political stress module—which has not yet undergone full psychometric validation—and show that country-specific stressors were significantly associated with overall psychological stress in this population, even if that stress was not reflected in the bruxism-related clinical findings studied here.

Age was one of the few variables that was independently linked to bruxism-related clinical findings: after correcting for relevant confounders, each additional year was associated with a 48.3% higher odds of bruxism-related clinical findings (OR = 1.483). Age is closely related to both academic year and the length of stay in Turkey. Because of this, the link with age may actually reflect the cumulative nature of symptoms such as tooth wear and muscle changes, rather than a direct effect of age itself. We cannot rule out cohort differences (differences between groups of people from different time periods). A longitudinal study is needed to find out if increasing age is truly associated with bruxism-related clinical findings, or if it simply reflects longer cumulative exposure to parafunctional activity (habits like teeth grinding or clenching).

Daily caffeine use was also independently associated with higher odds of bruxism-related clinical findings (OR = 4.12). However, smoking showed no significant association. Caffeine is known to stimulate the central nervous system. It may affect arousal and the activity of the chewing (masticatory) muscles. For this reason, a link between caffeine and bruxism is biologically plausible. However, because this study used a cross-sectional design, this association should not be seen as proof of a cause-and-effect relationship. Future longitudinal studies are needed to test whether caffeine use directly contributes to the start or the duration of bruxism.

Clinical findings related to bruxism were quite common in this group. This was especially true for tooth wear (78.8%) and abnormalities in soft tissue or masticatory muscles (72.6%). These frequencies were similar to those found in other studies of young adult populations [16,17].

However, it is impossible to determine if these results are unique to Iranian overseas students or rather represent the baseline prevalence of bruxism-related clinical characteristics commonly observed in young adults in the absence of a reference group. Future studies with appropriate comparison groups drawn from both domestic and other international student populations would help resolve this question.

Clinically, these findings suggest perceived stress alone should not be relied upon as a proxy for bruxism risk, and that broader psychosocial, behavioral, and duration-of-stay factors should be considered; the socio-political–perceived stress correlation nonetheless supports screening for home-country-related psychological burden in this population.

### Limitations of the Study

This pilot study has several limitations. First, sampling of academic years was uneven and non-random: coverage ranged from 74–100% among second- to fifth-year students, and first-year students could not be reached and are absent from the sample. Because age correlated with academic year, this gap may have introduced selection bias affecting the age–bruxism association observed, and findings should be considered hypothesis-generating rather than confirmatory. Second, this was a small, single-center study conducted at one Faculty of Dentistry, which limits generalizability beyond this specific population, and it lacked a non-Iranian (or non-international-student) comparison group; the absence of such a comparison group limits our ability to attribute the present findings specifically to socio-political stress rather than to international-student status more broadly. Participants may also have under-reported distress related to politically sensitive items despite anonymity safeguards, which would bias socio-political stress scores toward underestimation.

Third, although the socio-political stress module showed good post hoc internal consistency (α = 0.834) and exploratory evidence of internal coherence, it has not undergone full psychometric validation, including content validity, confirmatory factor analysis, or test–retest reliability. In addition, a possible ceiling effect was observed for the socio-political stress score, as 33 participants (29.2%) reached the maximum score and 64 participants (56.6%) scored ≥36 on the 0–40 scale. This restricted upper-end variability may have limited the ability to detect associations between socio-political stress and bruxism-related clinical findings. The Turkish PSS-10 also showed lower-than-expected reliability in this sample (α = 0.562); because the PSS-10 was administered in Turkish to non-native Turkish-speaking Iranian students, language- and culture-related response bias is a plausible contributor to this lower-than-expected reliability, which may in turn have attenuated its association with bruxism-related clinical findings.

Fourth, bruxism-related clinical findings status relied on a composite of self-report and clinical screening rather than instrumental criteria (e.g., polysomnography), although inter-examiner agreement was excellent for the primary outcome (κ = 0.982) and ranged κ = 0.900–1.000 across variables. Fifth, the cross-sectional design precludes causal inference; smoking and caffeine intake were adjusted for, but gender, anxiety, depression, sleep quality, and coping style were not measured. Larger, representative, longitudinal studies with comparison groups and a fully validated stress instrument are needed to confirm these findings.

Taken together, these findings suggest that clinical and university health services should not rely on perceived stress alone when screening for bruxism risk in international student populations, and should instead consider behavioral factors such as caffeine intake alongside psychological assessment. For future research, we recommend: instrumental confirmation of bruxism (e.g., electromyography or portable sleep monitoring) rather than reliance on clinical/self-report composites alone; development and validation of a Persian-language stress instrument tailored to this population; and longitudinal, multi-center designs incorporating a non-Iranian comparison group to disentangle international-student status from country-specific socio-political stress.

## 5. Conclusions

This study provides preliminary evidence that socio-political stress is closely related to perceived stress among Iranian university students studying in Turkey, highlighting the influence of home-country circumstances on the psychological well-being of international students. Even so, after adjusting for potential confounders, neither socio-political stress nor perceived stress was independently associated with bruxism-related clinical findings.

Instead, older age and higher daily caffeine intake were the variables independently associated with greater odds of bruxism-related clinical findings. These results highlight the multifactorial nature of bruxism and suggest that its clinical manifestations may owe more to cumulative behavioral or biological factors than to perceived stress measured at a single point in time. The dissociation observed here between psychological stress and bruxism-related clinical findings further suggests that stress-related pathways may not be fully captured by a cross-sectional assessment of either perceived stress or clinical signs.

These results should be interpreted cautiously due to the exploratory methodology and small sample size. To better understand the complex relationships between socio-political stress, psychological distress, and bruxism-related clinical findings, future research will need several improvements. This includes larger and more diverse groups of international students, suitable comparison groups, longitudinal follow-up (studying the same people over time), and validated tools that measure psychological stress and bruxism in a multidimensional way. Such studies could help identify people who might benefit from focused preventive and supportive interventions and expand our knowledge of the psychological factors that contribute to bruxism in populations of overseas students.

## Figures and Tables

**Table 1:** Characterization of the study participants.

Variable (*n*: 113)	Mean ± SD	Median	Min.–Max.
Age, years	24.1 ± 3.33	23	20–35
Gender	Female	67	59.3
	Male	46	40.7
Duration of residence in Turkey, months	50.1 ± 19.0	49	25–132
Academic year	3.29 ± 1.03	3	2–5
Socio-political stress total score	34.0 ± 7.44	36	0–40
PSS-10 total score	25.6 ± 4.71	24	12–37

**Table 2:** Internal consistency (Cronbach’s alpha) of study instruments.

Instrument	Number of Items	Cronbach’s Alpha	Corrected Item-Total Range	KMO	Bartlett’s Test	PCA Summary
Socio-political stress module	8	0.834	0.47–0.74	0.811	χ^2^ = 398.07, df = 28, *p* < 0.001	PC1 eigenvalue = 4.06; variance = 50.3%; loadings = 0.56–0.81
PSS-10 (Turkish version)	10	0.562	Reported in Supplementary Table S2	—	—	No clean single-factor solution

KMO and Bartlett’s test were reported for the researcher-developed socio-political stress module. PSS-10 item-level results are provided in Supplementary Table S2.

**Table 3:** Inter-examiner reliability (Cohen’s kappa) of bruxism-related clinical findings.

Clinical Variables (*n* = 113)	Cohen’s Kappa	Raw Agreement
BEWE score, right posterior	0.988	99.1%
BEWE score, anterior	0.958	95.6%
BEWE score, left posterior	0.963	97.3%
Maximum BEWE score	0.991	99.1%
Linea alba/Cheek biting marks/Tongue scalloping/Masseter hypertrophy/Bruxism-related clinical finding (primary outcome)	0.982	99.1%
Soft tissue/muscle finding ≥ 1	0.976	99.1%
Masseter palpation tenderness	0.938	92.9%
Temporalis palpation tenderness	0.969	96.5%
Lateral pterygoid palpation tenderness/Highest palpation score	0.994	99.1%
Jaw pain/locking	0.900	96.5%
Deviation during mouth opening	0.960	98.2%
Deviation direction/Joint sound/Tooth wear ≥ 1 (composite)	1.000	100.0%
Soft tissue/muscle finding ≥ 1 (composite)	0.935	97.3%
Muscle tenderness ≥ 1 (composite)	0.981	99.1%
TMD finding	0.943	97.3%

**Table 4:** Clinical and self-reported bruxism-related findings (*n* = 113).

(**a**). **Clinical examination findings.**			
**Variable**	**Category**	***n***	**%**
Bruxism-related clinical finding	Present	63	55.8
Daytime tooth clenching (0–4 scale)	Mean ± SD	—	2.10 ± 1.47
Morning jaw fatigue or headache (0–4 scale)	Mean ± SD	—	1.67 ± 1.42
Tooth wear ≥ 1 (BEWE)	Present	89	78.8
Soft tissue/muscle finding ≥ 1	Present	82	72.6
Muscle palpation tenderness ≥ 1	Present	72	63.7
TMD finding	Present	43	38.1
Jaw pain/locking	Present	26	23.0
Deviation during mouth opening	Present	37	32.7
Joint sound	Present	13	11.5
*For binary/present-absent variables, only the “present” row is shown; the complement (n, %) can be derived from the total (n = 113).* *Daytime tooth clenching and morning jaw fatigue/headache were rated on a 0–4 severity scale and are summarized as mean ± SD instead of full category breakdowns.*			
(**b**). **Self-reported findings.**			
**Variable**	**Category**	***n***	**%**
Sleep-related clenching/grinding reported by others	Yes	26	23.0
	No	41	36.3
	Do not know	46	40.7
Tooth sensitivity	Yes	41	36.3
Self-perceived tooth cracks/wear	Yes	28	24.8
*For Tooth sensitivity and Self-perceived tooth cracks/wear, only the “Yes” row is shown; the complement can be derived from the total (n = 113).*			

**Table 5:** Comparison of stress scores according to bruxism-related clinical findings status.

Variable	Bruxism-Related Clinical Findings Present (*n* = 63)	Bruxism-Related Clinical Findings Absent (*n* = 50)	Test Statistic	*p*-Value
Socio-political stress, median	37.0	36.0	Mann–Whitney U = 1542	0.849
PSS-10, median	25.0	24.0	Mann–Whitney U = 1558	0.921

Data are presented as medians because the distributions of the continuous stress variables departed from normality. Mann–Whitney U test was used as the primary between-group comparison.

**Table 6:** Correlation and logistic regression analyses.

Analysis	Variable/Predictor	Effect Estimate	95% CI	*p*-Value
Spearman correlation	Socio-political stress vs. PSS-10	rho = 0.339	0.16–0.49	<0.001
Fully adjusted logistic regression	Socio-political stress total score	0.972	0.906–1.044	0.442
Fully adjusted logistic regression	PSS-10 total score	0.966	0.868–1.075	0.526
Fully adjusted logistic regression	Age	1.483	1.203–1.828	<0.001
Fully adjusted logistic regression	Sex, female vs. male	1.723	0.639–4.644	0.282
Fully adjusted logistic regression	Caffeine intake (Moderate/high vs. none/low)	4.12	1.54–11.02	0.005
Fully adjusted logistic regression	Smoking status (Current smoker vs. non-smoker)	1.43	0.58–3.51	0.431

Smoking and caffeine intake were re-coded into binary categories to avoid assuming equal log-odds intervals across ordinal response categories. Odds ratios were adjusted for socio-political stress total score, PSS-10 total score, age, sex, smoking status, and caffeine intake. Effect estimates are presented as Spearman’s rho for correlation analysis and odds ratios for logistic regression.

## Data Availability

The de-identified individual-level data presented in this study are available on request from the corresponding author due to participant privacy and the politically sensitive nature of some survey items. De-identified aggregate data, the full analysis code, and the complete study instruments (sociodemographic questionnaire, socio-political stress module, and clinical examination form) are available from the corresponding author upon reasonable request.

## Supplementary Materials

The following supporting information can be downloaded at: https://www.mdpi.com/article/10.3390/healthcare14193224/s1, Table S1: Diagnostic assessment of the fully adjusted logistic regression model; Table S2: Item-level performance and component loadings of the socio-political stress module; Figure S1: Scree plot of the principal component analysis (socio-political stress module); Table S3: Inter-item correlation matrix of the socio-political stress module; Figure S2: Inter-item correlation heatmap of the socio-political stress module; Table S4: Principal component analysis of the socio-political stress module.

## Abbreviations

The following abbreviations are used in this manuscript:

BEWE	Basic Erosive Wear Examination
CI	Confidence Interval
OR	Odds Ratio
PCA	Principal Component Analysis
PC1	First Principal Component
PSS-10	10-Item Perceived Stress Scale
SD	Standard Deviation
TMD	Temporomandibular Disorder

## References

[1] Smith R.A., Khawaja N.G. (2011). A review of the acculturation experiences of international students. Int. J. Intercult. Relat..

[2] Forbes-Mewett H., Sawyer A.-M. (2016). International students and mental health. J. Int. Stud..

[3] Cohen S., Kamarck T., Mermelstein R. (1983). A global measure of perceived stress. J. Health Soc. Behav..

[4] Pedrelli P., Nyer M., Yeung A., Zulauf C., Wilens T. (2015). College students: Mental health problems and treatment considerations. Acad. Psychiatry.

[5] Lobbezoo F., Ahlberg J., Raphael K.G., Wetselaar P., Glaros A.G., Kato T., Santiago V., Winocur E., De Laat A., De Leeuw R. (2018). International consensus on the assessment of bruxism: Report of a work in progress. J. Oral Rehabil..

[6] Carra M.C., Huynh N., Lavigne G. (2012). Sleep bruxism: A comprehensive overview for the dental clinician interested in sleep medicine. Dent. Clin. N. Am..

[7] Manfredini D., Lobbezoo F. (2009). Role of psychosocial factors in the etiology of bruxism. J. Orofac. Pain.

[8] Rosales V.P., Ikeda K., Hizaki K., Naruo T., Nozoe S., Ito G. (2002). Emotional stress and brux-like activity of the masseter muscle in rats. Eur. J. Orthod..

[9] Winocur E., Uziel N., Lisha T., Goldsmith C., Eli I. (2011). Self-reported bruxism—Associations with perceived stress, motivation for control, dental anxiety and gagging. J. Oral Rehabil..

[10] Zareian Jahromi A., Brown J.L., Chhina Z., Rasmussen A. (2025). Sociopolitical events in the home country and psychological distress among Iranian immigrants in the United States: Investigating the role of emotion regulation, acculturation, and social support. J. Immigr. Minor. Health.

[11] Rahgozar S., Giménez-Llort L. (2024). Design and effectiveness of an online group logotherapy intervention on the mental health of Iranian international students in European countries during the COVID-19 pandemic. Front. Psychiatry.

[12] Eskin M., Harlak H., Demirkıran F., Dereboy Ç. (2013). The adaptation of the Perceived Stress Scale into Turkish: A reliability and validity analysis. New Symp. J..

[13] Manfredini D., Serra-Negra J., Carboncini F., Lobbezoo F. (2017). Current concepts of bruxism. Int. J. Prosthodont..

[14] Chemelo V.d.S., Né Y.G.d.S., Frazão D.R., Souza-Rodrigues R.D.d., Fagundes N.C.F., Magno M.B., Silva C.M.T.d., Maia L.C., Lima R.R. (2020). Is There Association Between Stress and Bruxism? A Systematic Review and Meta-Analysis. Front. Neurol..

[15] Akiba D., Perrone M., Almendral C. (2024). Study Abroad Angst: A Literature Review on the Mental Health of International Students During COVID-19. Int. J. Environ. Res. Public Health.

[16] Melo G., Duarte J., Pauletto P., Porporatti A.L., Stuginski-Barbosa J., Winocur E., Flores-Mir C., De Luca Canto G. (2019). Bruxism: An umbrella review of systematic reviews. J. Oral Rehabil..

[17] Manfredini D., Winocur E., Guarda-Nardini L., Paesani D., Lobbezoo F. (2013). Epidemiology of bruxism in adults: A systematic review of the literature. J. Orofac. Pain.

