# Supplementary material for: Association Between Socio-Political Stress, Perceived Stress, and Bruxism-Related Clinical Findings Among Iranian University Students Studying in Turkey: A Cross-Sectional Study
Source: Healthcare (Basel). 2026 Sep 30;14(19):3224. doi: 10.3390/healthcare14193224 (PMC13664811; doi:10.3390/healthcare14193224)
Supplement: Supplementary file 1 [file healthcare-14-03224-s001.zip › healthcare-4501717-supplementary.pdf]

Supplementary Materials

Association between Socio-Political Stress, Perceived Stress, and Bruxism-Related Clinical Findings among Iranian University Students Studying in Turkey: A Cross-Sectional Study

Supplementary Table S1. Diagnostic assessment of the fully adjusted logistic regression model

Diagnostic domain	Method	Result
Missing data	Complete-case evaluation	No missing data; *n* = 113 retained
Linearity in the logit	Box–Tidwell test for socio-political stress	$p = 0.164$
Linearity in the logit	Box–Tidwell test for PSS-10	$p = 0.458$
Linearity in the logit	Box–Tidwell test for age	$p = 0.427$
Overall logit-linearity	Joint Box–Tidwell test	$\chi^2 = 4.40$, df = 3, $p = 0.222$
Multicollinearity	Variance inflation factor	Maximum VIF = 1.34
Calibration	Hosmer–Lemeshow test	$\chi^2 = 12.84$, df = 8, $p = 0.117$
Discrimination	C-statistic / AUC	0.797
Discrimination uncertainty	Bootstrap 95% CI for AUC	0.705–0.878
Influential observations	Cook's distance	Maximum = 0.250; none >1.0
Conservative influence screening	Cook's distance >4/n	6 observations

Supplementary Table S2. Item-level performance and component loadings of the socio-political stress module

Item	Corrected item-total correlation	Alpha if item deleted	PC1 loading
Item 1	0.581	0.813	0.727
Item 2	0.554	0.820	0.658
Item 3	0.471	0.836	0.562
Item 4	0.503	0.832	0.598
Item 5	0.642	0.805	0.771
Item 6	0.601	0.812	0.757
Item 7	0.625	0.807	0.771
Item 8	0.741	0.796	0.814

PC1 loadings were obtained from principal component analysis of the eight socio-political stress items. All items loaded positively on the first component.

Supplementary Figure S1. Scree plot of the principal component analysis (socio-political stress module)

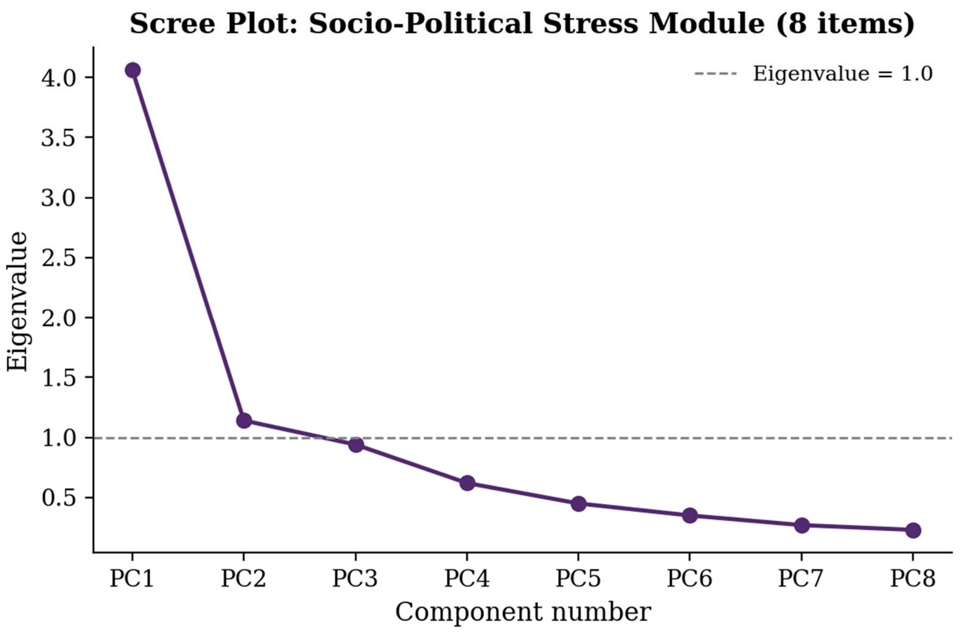

Eigenvalues for components 1-8, corresponding to Supplementary Table S4. The dashed line marks the conventional Kaiser criterion (eigenvalue = 1.0).

Supplementary Figure S2. Inter-item correlation heatmap of the socio-political stress module

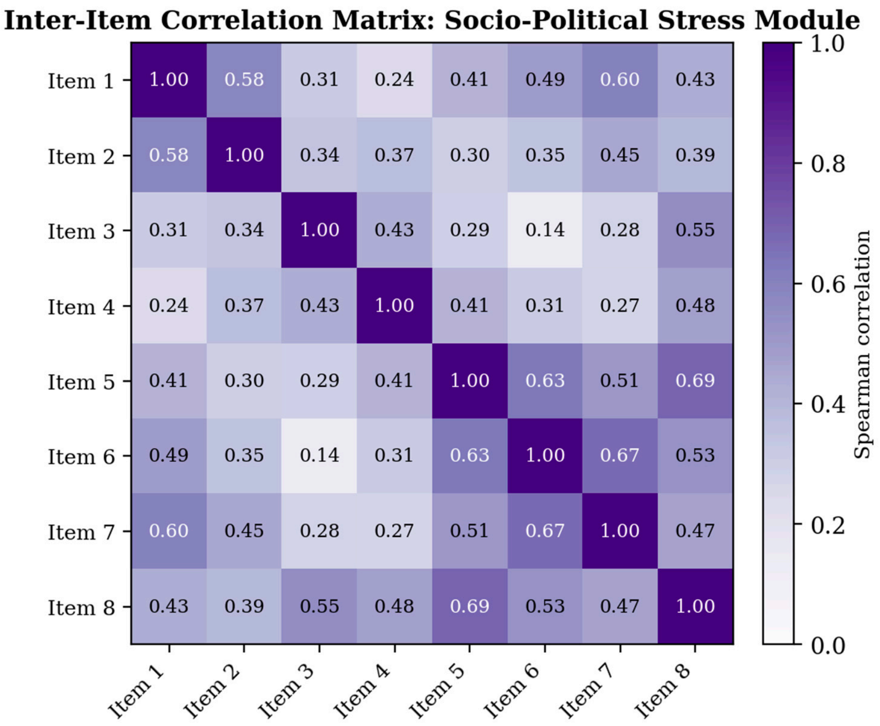

Graphical representation of the correlation matrix reported in Supplementary Table S3.

Supplementary Table S3. Inter-item correlation matrix of the socio-political stress module

	Item 1	Item 2	Item 3	Item 4	Item 5	Item 6	Item 7	Item 8
Item 1	1.00	0.58	0.31	0.24	0.41	0.49	0.60	0.43
Item 2	0.58	1.00	0.34	0.37	0.30	0.35	0.45	0.39
Item 3	0.31	0.34	1.00	0.43	0.29	0.14	0.28	0.55
Item 4	0.24	0.37	0.43	1.00	0.41	0.31	0.27	0.48
Item 5	0.41	0.30	0.29	0.41	1.00	0.63	0.51	0.69
Item 6	0.49	0.35	0.14	0.31	0.63	1.00	0.67	0.53
Item 7	0.60	0.45	0.28	0.27	0.51	0.67	1.00	0.47
Item 8	0.43	0.39	0.55	0.48	0.69	0.53	0.47	1.00

Supplementary Table S4. Principal component analysis of the socio-political stress module

Component	Eigenvalue	Variance explained, %	Cumulative variance, %
PC1	4.06	50.3	50.3
PC2	1.14	14.2	64.5
PC3	0.94	11.6	76.1
PC4	0.62	7.7	83.7
PC5	0.45	5.6	89.4
PC6	0.35	4.4	93.7
PC7	0.27	3.4	97.1
PC8	0.23	2.9	100.0

Although two components had eigenvalues >1.0, the scree plot and parallel analysis supported retention of one dominant component. Therefore, PCA findings were interpreted as exploratory evidence of internal coherence rather than definitive evidence of construct validity.
